# Supplementary material for: Design and evaluation of a pneumatic actuation unit for a wasp-inspired self-propelled needle
Source: PLoS One. 2024 Jul 2;19(7):e0306411. doi: 10.1371/journal.pone.0306411 (PMC11218968; doi:10.1371/journal.pone.0306411)
Supplement: Attachment — Submitted filename: Response to Reviewers.docx [file pone.0306411.s009.docx]

Dear Baeckkyoung Sung, Ph.D.,

Thank you for your time and consideration of our manuscript. Based on the reviewers’ comments below and your editorial comments, we have revised the manuscript accordingly.

Regarding the points described in your email, we confirm that (1) we adapted the manuscript to meet PLOS ONE’s style requirements, (2) we made all code available without restrictions, (3) we provided the grant numbers for our study in the ‘Funding Information’ section in the submission system and we added an updated financial disclosure statement in our cover letter, (4) we adjusted our ethics statement in Section 3.7 (Lines 368-370), and (5) we re-uploaded Supporting Information files [S1_Software_Arduino.txt and S3_SlipPneumaticOvipositorNeedle.txt] as text files.

Please find our detailed responses to the reviewers below. In the revised manuscript, the changes are indicated using track changes.

**Review Comments to the Author**

**Reviewer #1:** Thanks very much for this interesting work you are presenting. While being good to read, it lacks focus (is it the needle arrangement? is the pneumatic actuation? Is it MRI compatibility? Is it self-propelling insertion into liver?) and is lengthy in some parts (especially in the introduction), while you are citing a lot of other projects, it turns out that these are mostly from a group around Baena, by Scali or by your own group. A broader review is definitely necessary.

R1. Thank you for your compliments on the novelty of our work. Our pneumatic actuation unit works in unison with our developed wasp-inspired needle prototype, is MRI compatible, and can thus be used for a wide variety of medical applications. The focus is on the pneumatic actuation unit (see Lines 157-160).

Unfortunately, to our knowledge, the only groups working on wasp-inspired needles for friction manipulation to reduce tissue damage are Baena’s group at the Imperial College London and Breedveld’s group at the TU Delft. In previous work, we reviewed bio-inspired medical needles, which show other sources of bio-inspiration for percutaneous needles focused on different working principles, such as mosquito-inspired needles (Fung-A-Jou *et al.*, 2023) (Lines 81-82). Furthermore, we shortened the introduction section (Lines 88-92).

Fung-A-Jou Z, Bloemberg J, Breedveld P. Bioinspired medical needles: a review of the scientific literature. Bioinspiration & Biomimetics. 2023. doi: 10.1088/1748-3190/acd905.

The theoretic background should either be more comprehensive (preferable) or left out A single equation describing what a line of text said before is not useful. It would be interesting to know, how the difference of static and dynamic friction together with the stiffness of the needles, the stiffness of the tissue and – most important! – the strength of the tissue affect the movement. Also, the steerability (or straightness) of the needle’s movement must be assessed. Where could there be potential for improvement? How must the tips of the single needles be cut? How does the friction between the needles and the core (the laser fibre) affect its performance? How do fluids like blood (clotting?) or fat affect the performance in theory?

R2. We want to thank the reviewer for these useful suggestions. We, therefore, now elaborate on the in-text explanation of the different forces acting on the needle during insertion into tissue (Lines 100-141). In the explanation, we differentiated between the surface stiffness force in the pre-puncture phase and the cutting and friction forces in the post-puncture phase. Furthermore, we added information on how these different forces arise and by which factors they are affected. The self-propelled motion requires surrounding tissue. Thus, it works in the post-puncture phase where the surface stiffness force is equal to zero when assuming constant mechanical tissue properties in homogeneous tissue. In addition, as the needle propels deeper into the tissue, the surface area of the needle segments in direct contact with the tissue increases, increasing the friction forces on the needle segment linearly with insertion depth while the cutting force remains roughly constant throughout the insertion.

In the discussion in Lines 614-624, we elaborate on the integration of steering in the wasp-inspired needle to increase the effectiveness of the procedure and decrease the chance of unwanted tissue damage and pubic arch interference.

Secondly, we have added an explanation of the effect of integrating an optical fiber into the needle bundle's center on the needle's friction and cutting forces (Lines 575-587). We explain that in the two-phase motion sequence of the needle, retracting all six needle segments simultaneously advances the centrally positioned optical fiber into the substrate. The surface area of the needle segments in direct contact with the optical fiber is the same as the surface area of the needle segments in direct contact with each other in the current prototype. Consequently, we do not expect the optical fiber to influence the friction forces of the needle. However, the optical fiber does increase the cutting force during the retraction phase.

Thirdly, we added a paragraph discussing the foreseen challenges in moving towards *in vivo* testing (Lines 589-613). In this new paragraph, we discuss the effects of fluids like blood, which may reduce the needle-tissue friction required for the self-propelled motion. On the other hand, the parasitic wasp can move effectively through fluid-like substances like fruits thanks to directional friction patterns that increase friction. Inspired by these friction patterns, Parittotokkaporn *et al.* (2010) added a directional friction pattern to the needle surface. This could be an interesting avenue to explore for our needle in the future.

Parittotokkaporn T, Frasson L, Schneider A, Davies B, Degenaar P, y Baena FR. Insertion experiments of a biologically inspired microtextured and multi-part probe based on reciprocal motion. 2010 Annual International Conference of the IEEE Engineering in Medicine and Biology; 2010: IEEE. doi: 10.1109/IEMBS.2010.5627410.

If MRI-compatibility is important, I wonder how a 30 cm tube between your system and the valve block (which is electromagnetic) is MRI-compatible. But if you use longer tubes (like those 7 m between the nitrogen (why nitrogen, when you talk about air?) you will lose a lot of dynamics in the system. This should also be addressed and discussed.

R3. You are correct in that the valve block is currently not MRI-compatible. This is the reason why this part of the test set-up was strapped to a table. In the future, we intend to manufacture a pneumatic actuation unit with pneumatic valves and investigate fluidic logic, to prevent using electronics. Furthermore, we changed “air” in Line 381 to “nitrogen.”

Detailed issues

L40 type Trans blank Perineal

R4. Thank you for this suggestion. In Line 40, we have now changed TransPerineal to Trans Perineal.

L81 These refs are all from the same group. Is there nobody else on the planet working on these bio-inspired needles?

R5. Please refer to R1.

L103 This equation is just the beginning: how are F_cut and F_friction influenced?

R6. Please refer to R2.

L113 If other people have done that before, what is so new about your needle?  find good focus

R7. The novelty of our needle lies in the pneumatic actuation unit, please see Section 1.4.

L126 If electric motors are the problem for MRI, why not look a refs of people who used pneumatics?  it’s a question of focus the refs are all from the same group

R8. In our goal description, we describe current prototypes of wasp-inspired self-propelled needles. Wasp-inspired self-propelled needles using pneumatics do not exist yet in the scientific literature. Pneumatic actuation systems for general needles exist; they are mentioned in Lines 69-74.

L195 Tube length limits actuation frequency

R9. The effect of tube length on the airflow is discussed in Section 4.2, Lines 538-546.

L202 Quantify “minimally increasing” How thick is the shrink tube?

R10. Thank you for pointing out this missing information. In Section 2.4 in Line 228, we added the expanded inner diameter and wall thickness dimensions.

L214 what pressure are you envisioning? What frequency of actuation? How do you deal with leakage and noise?

R11. In the future, we would like to research the effect of different frequencies on the efficiency of the system. In the discussion, we added a line about investigating the implementation of bellows instead of pistons to prevent air leakage in the system (Lines 529-530). With future implementation, the effect of noise could also be investigated.

L222 please avoid first-person sentences and use passive voice.

R12. In Sections 3.1 and 3.7, Lines 249-250 and 362-366 were rewritten in passive voice.

L233 what friction coefficients are you expecting?

R13. The friction coefficient depends on the exact tissue properties in which we test the device. At the moment, we have not performed any experiments to measure this friction coefficient. In the discussion in Lines 594-596, we added that directional friction patterns, as investigated by Parittotokkaporn *et al*. (2010), could be an interesting avenue to explore.

Parittotokkaporn T, Frasson L, Schneider A, Davies B, Degenaar P, y Baena FR. Insertion experiments of a biologically inspired microtextured and multi-part probe based on reciprocal motion. 2010 Annual International Conference of the IEEE Engineering in Medicine and Biology; 2010: IEEE. doi: 10.1109/IEMBS.2010.5627410.

L241 here, you use 0.5 bar in L346 you talk about 3 bar. What is true or why is it different?

R14. Based on the experiments in gelatin samples we decided to increase the pressure for the *ex vivo* tissue experiments to improve the cutting capability of the needle.

L258 If the experimental setup dates back to 2019 what has happened ever since?

R15. It is true that the experimental setup used in this study is similar to the setup described in 2019. However, the design, as explained in Section 2, evaluated with the experimental setup has changed.

L267 in my opinion, 300 m distance between needle and valvle block are far too short for MRI compatibility!

R16. Please refer to R3. MRI compatibility in this research mainly involves the actuation unit parts and other parts crucial to the experimental setup.

L277 young’s modulus is of inferior interest here. The strength of the material and the friction are of most importance!

R17. For the working principle of the wasp-inspired needle, the friction coefficient and cutting force are most important during propulsion. However, because we wanted the gelatin phantoms to mimic mechanical tissue properties, we indicated Young’s modulus of the gelatin phantoms.

L334 a demonstration is nice but should be described much shorter. As an alternative this could be a real experiment where you show how straight your needle goes, how little the image distortions are how fast you go etc. But not like “see, it works!”

R18. Thank you for your suggestion. We shortened the text about the additional MRI experiment and removed it as a separate Section (i.e., Section 4. MRI experiment). Instead, we added it as a subsection to Section 3. Performance evaluation. The new subsection is called “3.7 Additional MRI experiment”.

L340 again: young’s miodulus is not the point, strength and friction are more important.

R19. Please refer to R17.

L361 if you refer to your previous work, make clear what is new about this here

R20. Solely the low-friction design inside the RF coil of the pre-clinical MRI scanner was the same as the low-friction design used in our previous work. We also clarified this in the manuscript in Lines 390-391.

L363 do you use air or nitrogen?? Please be precise!

R21. Thank you for pointing out this discrepancy. In Section 3.7 (Line 381) air was replaced by nitrogen.

L368 Liver has a density around 1.079 g/cm³ why is your 45 cm³ piece so heavy?

R22. Thank you for pointing out this typo. The weight of the 45 cm^3^ liver-agar sample was 46 grams. We adjusted the text accordingly in Section 3.7 (Line 398).

L415 The discussion is far too long. Please focus on your main research question and do not discuss future projects. This is a paper not a Master’s thesis!

R23. Thank you for this suggestion. We removed the discussion on the future project regarding the control of the needle.

L559 55 ref are more than enough…

R24. In our opinion, these references are essential to underpin the claims in our manuscript, ensuring the reliability of the information presented.

Fig 1 give the reader an impression of size do not rely on colours; Fig 2 also misses a scale; Fig 5 b misses a scale; Fig 9 a misses a scale; Fig 10 b misses a scale.

R25. Thank you for these suggestions. To give the reader an impression of the size in the images, we have added scale bars in Figs 1, 2b, 5b, 9abc, 10c.

**Reviewer #2:** The paper proposes a novel self-propelled needle designed for MRI-guided percutaneous laser ablation of the prostate. The self-propelling mechanism is inspired by the wasp’s ovipositor and consists of six needle segments that are driven by pneumatic actuators independently. The newly developed needle was tested in a gel phantom and an ex vivo tissue. While the mechanism has been proposed previously, this work appears to be the first attempt to make such a mechanism work in the MRI environment.

The work offers a solid contribution to the research community. If successful, the proposed mechanism will allow placing a needle into the prostate under live MRI guidance more efficiently, as it addresses the issues associated with MRI guidance, such as the narrow workspace in the MRI gantry and the deviation of the needle due to tissue-needle interaction. The paper is well written with sufficient detail of the work.

However, the performance of the self-propelling mechanism, especially the slip ratio, is somewhat unconvincing, probably due to the lack of comprehensive understanding of the forces involved in the propelling. This reviewer still thinks that the experimental data are worth publishing, though the paper needs to address the following questions.

(1) (Major) Equation (1) only considers the cutting force and the friction between the needle shaft and the tissue, though it is not clear if they are the dominant forces when considering the propulsion of the needle. If the friction is dominant, the slip ratio in Figure 7 should have increased as the needle advances into the tissue because the friction should be proportional to the contact area (i.e., the length of the needle inside the tissue). However, the figures do not seem to show such an effect except S4-105.

R26. Thank you for your compliments. We extended Eq. 1 with the surface stiffness force. Please refer to R2 above. Indeed, the friction is length-dependent; as the needle propels deeper into the tissue, the surface area of the needle segments in direct contact with the tissue increases, increasing the friction forces on the needle segment linearly with insertion depth while the cutting force remains roughly constant throughout the insertion.

At the moment, the slip ratio of the needle in the tissue (phantom) is relatively high, which makes it difficult to observe the effect of friction increase during needle insertion. However, in theory, this effect would be beneficial for the self-propelling motion of the needle.

(2) Related to comment 1, did the authors measure the friction and cutting forces independently? They could have measured them by inserting and retracting the needle in the phantom while measuring the force along the needle. (Assuming that the thrust force required for insertion would equal the sum of the friction and the cutting force, while that for retraction would only equal the friction.)

R27. The friction forces and cutting forces were not measured in the experiments. However, we agree that this is an interesting experiment to perform to determine the cutting and friction forces acting on a single needle segment in the future. In Section 4.3 we added a line about this future investigation (Lines 587-588)

(3) Again related to comment 1, the authors listed the inertia and friction of the cart as limitations in section 5.2. If the slip ratio is not clearly influenced by the insertion length, wouldn’t it be more reasonable to consider those factors in Equation (1)? They are independent of the insertion length. If the authors measured the displacement of the cart, they could have estimated the force from the inertia based on the mass and the acceleration of the cart.

R28. Thank you for your valuable suggestion. In Section 4.2 in Lines 531-537, we discuss that the mass of the cart and the gelatin phantom or tissue tends to increase the slip in the retracting phase of the needle segments, whereas the contrary holds for the advancing phase. In the retracting phase, the resultant force on the needle inside the tissue (phantom) is likely to be lower than the force required to overcome the bearing friction and cart inertia. Therefore, the effect of inertia and bearing friction cannot be ruled out.

(4) (Moderate) The authors only consider the material property of the prostate, but the prostate tissue might be a small portion of the overall insertion path, probably ~20-30mm out of ~100mm insertion in the transperineal insertion. The rest consists of tissues with significantly different properties, such as muscles, skin, etc. The presented experiment is a good first step, but it would be helpful if the authors discussed what the results mean for other types of tissues (e.g., whether harder/softer tissue results in higher/lower friction ratio).

R29. Thank you for your suggestions. In the discussion, we added a paragraph (Lines 589-613 discussing the foreseen challenges in moving towards *in vivo* testing. In this new paragraph, we discuss the effects of more and multi-layered tissue with different properties between the needle insertion point in *in vivo* conditions (i.e., the perineum) and the target point inside the prostate.

(5) (Moderate) The authors discuss air leakage as one of the limitations. Have they considered diaphragms or bellows in place of pistons? There were studies used bellows for actuation, such as: Comber DB, IEEE Trans Robot. 2016 Feb;32(1):138-149. doi: 10.1109/TRO.2015.2504981. Epub 2016 Jan 19. PMID: 31105476; PMCID: PMC6519471.

R30. Thank you for this suggestion. In the discussion in Lines 529-530, we have now added a suggestion to investigate the use of bellows for future versions of the Pneumatic Ovipositor Needle.

(6) (Minor) Is the dimensions of the needle large enough to accommodate the optic fiber for laser ablation? What is the authors’ plan to scale up this prototype to incorporate the ablation device?

R31. Optical fibers for TPLA have an outer diameter of 300 µm. Integrating a 300-µm diameter optical fiber into the center of the needle bundle consisting of six 0.25-mm diameter needle segments results in a total needle bundle outer diameter of 0.8 mm. So, this would still fit within the shrink tube with an inner diameter of 0.814 mm and an outer diameter of 0.84 mm. In Section 4.3 Lines 576-587, we added the dimensions of the TPLA fiber and how it could be accommodated in the needle.

**Reviewer #3:** It is a well-written paper and I suggest the paper to be accepted with a revision. I think the discussion on the setup for the clinical setting needs to be presented in more details. I understand the limitation of the in-lab experiment however I would like to see further insights on how to do the actuation in a clinical setting. A different actuation method may completely result in a different conclusion. Could a rough experiment be performed?

R32. Thank you for your compliments. In the discussion, we added a paragraph discussing the foreseen challenges in moving towards *in vivo* testing (Lines 589-613). In this new paragraph, we discuss the effects of more and multi-layered tissue between the needle insertion point in *in vivo* conditions (i.e., the perineum) and the target point inside the prostate. Other non-pneumatic self-propelled needles have been shown to be able to self-propel in multi-layered tissue-mimicking phantoms (Scali *et al.*, 2019). Moreover, as the needle is inserted further into the tissue, the role of the cutting force on the single advanced segment becomes less pertinent, which, in theory, would aid the self-propelling motion of the needle. Lastly, in this study, we kept the actuation unit stationary while the tissue was placed on a low-friction support structure that moved toward the needle. In clinical practice, the patient will have to remain stationary while the needle self-propels inside the tissue. To accommodate this, the needle could be moved inside the actuation unit to manipulate the needle towards the patient, following the pace of the self-propelling motion of the needle.

Scali M, Breedveld P, Dodou D. Experimental evaluation of a self-propelling bio-inspired needle in single-and multi-layered phantoms. Scientific reports. 2019;9(1):1-13. doi: 10.1038/s41598-019-56403-0.

Thank you for your useful suggestions. We look forward to your reaction.

With kind regards,

Jette Bloemberg and co-authors
