# Supplementary material for: Design and evaluation of a pneumatic actuation unit for a wasp-inspired self-propelled needle
Source: PLoS One. 2024 Jul 2;19(7):e0306411. doi: 10.1371/journal.pone.0306411 (PMC11218968; doi:10.1371/journal.pone.0306411)
Supplement: Attachment — Submitted filename: Response to Reviewers.docx [file pone.0306411.s010.docx]

Dear Baeckkyoung Sung, Ph.D.,

Thank you for your time and consideration of our manuscript. Based on the reviewers’ comments below and your editorial comments, we have revised the manuscript accordingly. Please find our detailed responses to the reviewers below. In the revised manuscript, the changes are indicated using track changes.

**Review Comments to the Author**

**Reviewer #1:** Thanks very much for this thorough revision of your paper. It reads much better, while still is very long. The focus is a bit clearer now.

R1. Thank you for your compliments on our revised manuscript. Regarding the length of the manuscript, please refer to R5.

You introduced a stiffness force in the equilibrium equation. However, this is dependent on the actual movement (F=k*x) and rising with stroke while the other forces are more or less constant (F_cut) or dependent on the insertion depth (F_fric). I understand that F_stiff is acting in the direction of movement, because otherwise it would be F_fric. Please explain.

R2. In Equation 1, we introduce the surface stiffness force, the friction force, and the cutting force. Both the surface stiffness force and the cutting force arise at the needle tip, whereas the friction force occurs along the length of the needle. The surface stiffness is due to the elasticity of the tissue layer and occurs when puncturing the skin or a stiffer tissue layer than the current surrounding tissue, e.g., when puncturing the membrane around an organ. We extended the explanation of the surface stiffness force in Line 103. The cutting force arises at the needle tip and is, amongst others, caused by the needle still encountering stiffness as it cuts through the tissue. We clarified the explanation of the cutting force in Lines 110-111.

Concerning you R17: I must insist that Young’s modulus is of lesser interest when characterizing the cutting or ripping properties of a tissue than the actual material strength. Young’s modulus is a material-specific elasticity, not a strength. It would show you how elastic a tissue behaves, but not how much force you need to cut it. Let me make a comparison: Aluminium (no matter which alloy) has a Young’s modulus of about 70000 N/mm² while steel has roughly 210000 N/mm². A simple construction steel (S235, 1.0038) has a maximum strength of 360 N/mm² while a high-end aluminium alloy like AlZnMgCu1,5 can bear up to 540 N/mm². Which material is stronger, which is more elastic?

R3. Thank you for your valuable suggestion. We agree with you that for evaluation of the propulsion of the needle inside tissue (phantoms), mechanical properties, such as the needle-tissue friction and the tissue (phantom) cutting and ripping properties, e.g., the ultimate strength of the tissue, are of higher interest than the Young’s modulus of the tissue (phantom). However, to our knowledge, the majority of the mechanical properties of human tissues, such as needle-tissue (phantom) friction and tissue (phantom) ultimate strength are not described in the scientific literature. Thus, we chose to report the known mechanical properties of the prostate tissue and the gelatin phantoms, namely the modulus of elasticity (Kelly et al., 2019). We clarified this in Lines 303-306.

In addition, in the discussion in Lines 478-483, we added the recommendation to investigate the mechanical properties of prostate tissue and the corresponding tissue-mimicking materials important for needle insertion and propulsion, such as the needle-tissue friction coefficient and tissue ultimate strength.

- Kelly NP, Flood HD, Hoey DA, Kiely PA, Giri SK, Coffey JC, et al. Direct mechanical characterization of prostate tissue—a systematic review. The Prostate. 2019;79(2):115-25. doi: 10.1002/pros.23718.

About the state of the art. I still feel some kind of unhappy with the situation that there seem to be such limited number of groups working on that field. I found the group of Oliver Schwarz from Stuttgart. He is working on pneumatically-assisted tools based on the Sirex wasp ovipositor. However, these seem to be bigger than your device. In addition, I found https://doi.org/10.1177/09544119221137133, DOI 10.1088/1748-3190/acfb65 (it is SMA-actuated…)

R4. Thank you for your suggestions. In the introduction, we added the references to the mosquito-inspired needles of Acharya *et al.* (2023) and Gidde *et al.* (2023) in Line 82 where we state that in the scientific literature, other sources of bioinspiration, such as mosquitoes, were used for needles that employ different working principles.

- Acharya SR, Hutapea P. An experimental study on the mechanics and control of SMA-actuated bioinspired needle. Bioinspiration & Biomimetics. 2023;18(6):066008. doi: 10.1088/1748-3190/acfb65.
- Gidde STR, Islam S, Kim A, Hutapea P. Experimental study of mosquito-inspired needle to minimize insertion force and tissue deformation. Proceedings of the Institution of Mechanical Engineers, Part H: Journal of Engineering in Medicine. 2023;237(1):113-23. doi: 10.1177/09544119221137133.

Furthermore, in Lines 83-84, we have added that wasp-inspired propulsion has also been shown to be useful on a larger scale in a drilling device for medical applications (Nakajima and Schwarz, 2014).

- Nakajima K, Schwarz O. How to use the ovipositor drilling mechanism of Hymenoptera for developing a surgical instrument in biomimetic design. International Journal of Design & Nature and Ecodynamics. 2014;9(3):177-89. doi: 10.2495/DNE-V9-N3-177-189.

The length of the paper is still exhausting. Especially the discussion (including limitations and recommendations is far too much. Please cut down to a max of 1.5 pages. This should not be a summary but a discussion.

R5. Thank you for your suggestion. In response, we have streamlined our discussion by omitting the results of our experiments and the discussion slip ratio peaks in Section 4.1 Main findings. Additionally, we have removed the discussion on longer stroke lengths from Section 4.2 Limitations. Lastly, we condensed the content of Section 4.3 Recommendations and Future Research.

**Reviewer #2:** All the previous comments from this reviewer have been addressed. The manuscript is recommended for publication.

R6. Thank you for your kind words.

Thank you for your useful suggestions. We look forward to your reaction.

With kind regards,

Jette Bloemberg and co-authors
