# Supplementary material for: Design and evaluation of a pneumatic actuation unit for a wasp-inspired self-propelled needle
Source: PLoS One. 2024 Jul 2;19(7):e0306411. doi: 10.1371/journal.pone.0306411 (PMC11218968; doi:10.1371/journal.pone.0306411)
Supplement: Attachment — Submitted filename: Response to Reviewers .docx [file pone.0306411.s011.docx]

Dear Baeckkyoung Sung, Ph.D.,

Thank you for your time and consideration of our manuscript. Based on the reviewer’s comments below, we have revised the manuscript accordingly. Please find our detailed responses to the reviewer below. In the revised manuscript, the changes are indicated using track changes.

**Review Comments to the Author**

Reviewer #1: Thanks again very much for the diligent revision of your paper. It has again improved, while it is still very long.

R1. Thank you for your compliments on our revised manuscript. Regarding the length of the manuscript, please refer to R4.

You cite Abolhassani 2007 with his paper on friction forces on needles. Why not have a drawing that illustrates the forces acting on the needle instead of relying on a simple equation. Abolhassani also gives some info on friction and stiffness in eqs. 2 and 3. As your device is strongly relying on friction this is worth a try.

R2. In Figure 2A, we visualized the ovipositor-inspired needle using a schematic drawing. The drawing shows a two-dimensional illustration of ovipositor-inspired needle insertion into tissue. In the drawing, we illustrated the forces acting on the needle, i.e., the friction force along the advancing needle segment, the cutting force on the tip of the advancing needle segment, and the friction force along the retracting needle segments, which works in the opposite direction as the friction force of the advancing needle segments.

Material parameters could be estimated from these papers:

* Ankersen 1999 - Puncture resistance and tensile strength of skin simulants

* Gallagher 2012 - Dynamic Tensile Properties of Human Skin (they also give some literature overview)

* Johnson 2021 - Characterizing the Material Properties of the Kidney and Liver in Unconfined Compression and Probing Protocols with Special Reference to Varying Strain Rate (Fig 5)

* Kelly 2018 - Direct mechanical characterization of prostate tissue—a systematic review (promising title, but no real info on strength, as they use elastography…)

* Kemper 2010 - Biomechanical Response of Human Liver in Tensile Loading

* Liang 2016 - Simulation and experiment of soft-tissue deformation in prostate brachytherapy (at least some info on shear modulus)

* Wang 2014 - Alterations in mechanical properties are associated with prostate cancer progression (material stregth only to a certain extend)

R3. Thank you for your suggestions on estimating the material parameters of tissue. We agree that for evaluating the propulsion of the needle inside tissue phantoms and *ex vivo* specimens, mechanical properties, such as the needle-tissue friction, shear modulus, and ultimate strength, are of interest. However, the material parameters of the skin, such as its puncture resistance (Ankersen, 1999) and its dynamic tensile properties (Gallagher, 2012), are not of interest to our needle in the current evaluation stage because the self-propelled motion of our ovipositor-inspired needle requires contact with surrounding tissue and is not used during the puncture phase. Therefore, it works in the post-puncture phase where the surface stiffness force, i.e., the pre-puncture force due to the elasticity of the skin layer, equals zero. Consequently, we evaluated our needle’s slip ratio after an initial insertion distance of 35 mm where the surface stiffness force does not play a role.

Nevertheless, we agree that the mechanical properties of tissue phantoms and *ex vivo* tissue specimens, such as the needle-tissue friction, shear modulus, and ultimate strength, are of interest. We clarified this in Lines 306 and 436-443 of our revised manuscript. Furthermore, when using *ex vivo* porcine tissue specimens, we added the important remark that material properties may vary from human tissue (Johnson, 2021).


To solve this argument, you could either focus the paper more on the technical aspect of *building* the device and leave out the theory background or neglect the building (and being happy that it works) and focus on the theory *why* it works.

The length of the paper is still above average.

R4. According to the submission guidelines of PLOS ONE, there are no restrictions on word count for a manuscript. In our opinion, the background theory and the technical aspects of developing and producing the design are essential for the thoroughness and completeness of our manuscript.

Thank you for your valuable suggestions. We look forward to your reaction.

With kind regards,

Jette Bloemberg and co-authors
