# Supplementary material for: Design and evaluation of a pneumatic actuation unit for a wasp-inspired self-propelled needle
Source: PLoS One. 2024 Jul 2;19(7):e0306411. doi: 10.1371/journal.pone.0306411 (PMC11218968; doi:10.1371/journal.pone.0306411)
Supplement: Attachment — Submitted filename: Response to Reviewers.docx [file pone.0306411.s012.docx]

Dear Baeckkyoung Sung, Ph.D.,

Thank you for your time and consideration of our manuscript. Based on the reviewer’s comments below, we have revised the manuscript accordingly. Please find our detailed responses to the reviewer below. In the revised manuscript, the changes are indicated using track changes.

**Review Comments to the Author**

Reviewer #1: Thanks again very much for the new revision of your paper. It is still very long. I agree that there is no page limit by PLOS1 but I am convinced, that keeping a paper as brief as possible is a quality. There is not much “background theory” in your paper apart from eq. 1. Also, you do not compare theoretically derived values (Forces, Stroke etc.) with those you measured (eg. Fig. 8). Thus, I suggest for the sake of brevity to skip the “background theory” – or provide a real model describing the expected behaviour of your device… Don’t get me wrong: you built a great device. Wonderful engineering and outstanding experimental work. But you fail to explain, why it works the way it does quantitatively. Anyway. Keep it as is. Reviewers shouldn’t argue with authors and vice versa.

R1. Thank you for your compliments on our revised manuscript.

In eq. 1 you list all forces acting on the needle while in Fig. 2a F_{stiff} is missing. In your rebuttal you explain that this force is not necessary as the needle acts only deep in the tissue and the stiffness would act only while puncturing the tissue. I found this explanation on your manuscript in lines 103 ff but not in connection with eq. 1. Consequently, the reader would miss F_{stiff}. In addition. As the tissue is elastic AND friction acts, I would assume that after most of the accumulated F_{stiff} is released after puncturing the surface, there would still be a residual part of F_{stiff} acting. But I might be wrong. Still, if F_{stiff} appears in eq. 1 it must be defined or explained in Fig 2a.

R2. Thank you for your suggestion. In the figure caption of Fig 2A, we added that this is an illustration of the needle in the post-puncture phase after the needle has penetrated the outer tissue layer, therefore, $\mathbf{F}_{\mathrm{stiff}}$ is not present. Furthermore, in Lines 107-109 and 131-132, we explained that $\mathbf{F}_{\mathrm{stiff}}$ is only present when puncturing tissue layers. After the needle has punctured the tissue layer, $\mathbf{F}_{\mathrm{stiff}}$ returns to zero whilst self-propelling through homogeneous tissue.

Thank you for your valuable suggestions. We look forward to your reaction.

With kind regards,

Jette Bloemberg and co-authors
